# The Toxic Filter: Chronic Kidney Disease Drives Neuro‐Psychiatric Toxicity and Agitation in Sepsis Survivors Aged ≥ 90 Years: A Retrospective Cohort Study Using the National Database of Japan

**DOI:** 10.1111/psyg.70191

**Published:** 2026-07-07

**Authors:** Yuichiro Shimoyama, Noriko Kadono, Osamu Umegaki

**Affiliations:** ^1^ Department of Anesthesiology Osaka Medical and Pharmaceutical University, Intensive Care Unit, Osaka Medical and Pharmaceutical University Hospital Takatsuki Osaka Japan

**Keywords:** chronic kidney disease, metabolic dichotomy, patients aged ≥ 90 years, post‐intensive care syndrome, toxic agitation

## Abstract

**Background:**

In sepsis survivors aged ≥ 90 years, physiological reserve defines recovery. We previously identified liver dysfunction as protective (‘liver paradox’) and rehabilitation as a trigger for mental decline. Here, we hypothesised a ‘metabolic dichotomy’, where chronic kidney disease (CKD) damages the brain by accumulating neurotoxins, acting as a ‘toxic filter’.

**Methods:**

Using the National Database of Japan, we analysed 269 previously independent and medication‐free sepsis survivors aged ≥ 90. The primary outcome was new initiation of CNS‐active medications within 6 months after ICU admission. A secondary analysis compared initiation of antipsychotics (agitation/delirium) versus antidementia drugs. We performed multivariate logistic regression adjusting for confounders including rehabilitation.

**Results:**

Among 269 survivors, 49 (18.2%) had CKD. The CKD group had significantly higher antipsychotic initiation (34.7% vs. 19.1%; *p* = 0.022), whereas antidementia drug initiation was identical (4.1% vs. 4.1%; *p* = 1.00). Multivariate analysis identified CKD as an independent risk factor for overall CNS medication initiation (OR 2.85; *p* = 0.009). Specifically for antipsychotics, CKD's impact was more pronounced (OR 3.22; *p* = 0.004). Sensitivity analyses excluding renal replacement therapy yielded consistent results (OR 3.21; *p* = 0.006). Liver dysfunction maintained a protective trend.

**Conclusion:**

Unlike the protective liver, CKD acts as a ‘toxic filter’, driving acute ‘toxic agitation’ rather than dementia in nonagenarians. This establishes the ‘metabolic dichotomy’. Preserving renal filtration is synonymous with preserving sanity.

## 1 Introduction

### 1.1 The Nonagenarian Puzzle: Body, Brain and Metabolism

As rapid global population ageing progresses, the provision of intensive care for nonagenarians (aged ≥ 90) has become a critical ethical and clinical challenge [1, 2, 3]. While advancements in critical care medicine have improved short‐term survival rates, the quality of survival often deteriorates, leading to the devastating condition known as post‐intensive care syndrome (PICS) [4, 5]. Through our comprehensive series of studies on previously independent and medication‐free survivors, we have mapped the trajectory of their survival and identified key physiological determinants that separate resilience from collapse:

1. *The body*: We discovered the ‘liver paradox’, where reduced hepatic metabolism paradoxically protects against iatrogenic drug overdose and preserves physical independence [1].
2. *The brain*: We demonstrated that general anaesthesia creates a latent neuro‐vulnerability, shifting the clinical phenotype towards sleep‐sensory dysregulation rather than frank dementia [6].
3. *The process*: We revealed that ‘active rehabilitation’ can trigger neuro‐psychiatric exhaustion in those with limited metabolic reserve, a phenomenon we termed ‘the rehabilitation paradox’ [7].

### 1.2 The Missing Link: The Kidney

However, a critical piece of the metabolic puzzle remains missing: the kidney. While the liver and kidney are both central to pharmacokinetics and systemic homeostasis, their functions in the context of critical illness are diametrically opposed—metabolism (liver) versus excretion (kidney). The ‘liver paradox’ suggests that *slowing down* metabolic breakdown is protective by preventing excessive sedation and energy expenditure. In contrast, does the *stopping* of excretion by the kidney act as a fatal blow? The kidney is not only responsible for fluid and electrolyte balance but is the primary route for eliminating water‐soluble toxins, active drug metabolites and inflammatory cytokines [8]. In nonagenarians, whose blood–brain barrier is already compromised by age and sepsis‐induced inflammation, the failure of this filtration system could have catastrophic consequences for the central nervous system [9].

### 1.3 Hypothesis: The ‘Metabolic Dichotomy’

We propose the ‘metabolic dichotomy’ hypothesis. In the fragile brain of nonagenarians, we postulate that chronic kidney disease (CKD) acts not merely as a marker of disease severity or vascular burden, but as a ‘toxic filter’ that actively accumulates uremic toxins and active drug metabolites. Specifically, we hypothesise that this accumulation manifests clinically not as the slow, progressive cognitive decline typical of Alzheimer's‐type dementia, but as acute ‘toxic agitation’ (characterised by delirium, excitement, restlessness and behavioural disturbances), necessitating antipsychotic intervention [10]. This study aims to complete our physiological map of the nonagenarians by contrasting the ‘protective liver’ with the ‘toxic kidney’, utilising a massive nationwide dataset to test this hypothesis.

## 2 Methods

### 2.1 Study Design and Population

This retrospective cohort study utilised the National Database of Japan (NDB), which covers approximately 98% of all medical claims in Japan [11, 12]. We identified sepsis patients aged ≥ 90 admitted to ICUs claiming the specific intensive care unit management fee between 2015 and 2020. Sepsis was defined based on ICD‐10 codes for bacterial sepsis (A40.x, A41.x) or fungal sepsis (B37.7). From this population, we identified a strictly selected cohort of 269 previously independent and medication‐free survivors. This cohort was defined as patients who had no history of home medical care, rehabilitation or psychotropic medication use (antipsychotics, antidepressants, anxiolytics antidementia drugs) in the 6 months prior to ICU admission. This strict selection eliminates baseline confounding by pre‐existing frailty, dementia or psychiatric conditions [1, 6, 7].

### 2.2 Ethical Approval

This study was approved by the Ethics Committee of Osaka Medical and Pharmaceutical University and the Ministry of Health, Labour and Welfare of Japan for the use of NDB data. The requirement for informed consent was waived due to the anonymized nature of the data.

- Variables
  ○ *Exposure*: CKD was defined based on ICD‐10 codes (N18.x) recorded in the claims data. We also collected data on other comorbidities, including liver dysfunction, which was defined using the standard ICD‐10 codes for diseases of the liver (K70–K77). Data on hypertension, diabetes, dyslipidaemia and therapeutic interventions were also collected.
- Outcomes
  ○ *Primary outcome*: The new initiation of any CNS‐active medications (antipsychotics, antidepressants, anxiolytics, antidementia drugs, hypnotics) within 6 months after ICU admission.
  ○ *Secondary outcome (phenotype analysis)*: To discern the nature of the neuro‐psychiatric decline, we specifically analysed the initiation of antipsychotics (indicating agitation/delirium) compared with antidementia drugs (indicating cognitive decline).

### 2.3 Statistical Analysis

We employed Fisher's exact test for univariate comparisons. For multivariate analysis, we constructed logistic regression models to identify independent predictors. To rigorously isolate the independent impact of the ‘toxic filter’ (CKD), our multivariate models a priori incorporated the key physiological determinants identified in our preceding series of studies (Reports 1–3): Liver dysfunction (the protective shield) [1], general anaesthesia (the neuro‐vulnerability) [6] and rehabilitation (the metabolic trigger) [7], along with age. Sensitivity analyses excluding patients who underwent continuous haemodiafiltration (CHDF) were also performed. In accordance with NDB privacy protection regulations, cells containing fewer than 10 patients are suppressed or denoted as ‘< 10’. Furthermore, specific demographic identifiers, such as sex, were completely masked across all groups to eliminate any theoretical risk of patient re‐identification within this highly restricted cohort of patients aged ≥ 90 years. All statistical analyses were performed using JMP Pro software version 11.0.0 (SAS Institute Inc., Cary, NC, USA). A *p*‐value < 0.05 was considered statistically significant. We assessed multicollinearity using the variance inflation factor (VIF) to ensure the independence of covariates (all VIFs were < 10).

## 3 Results

### 3.1 Study Population Selection

**FIGURE 1:**
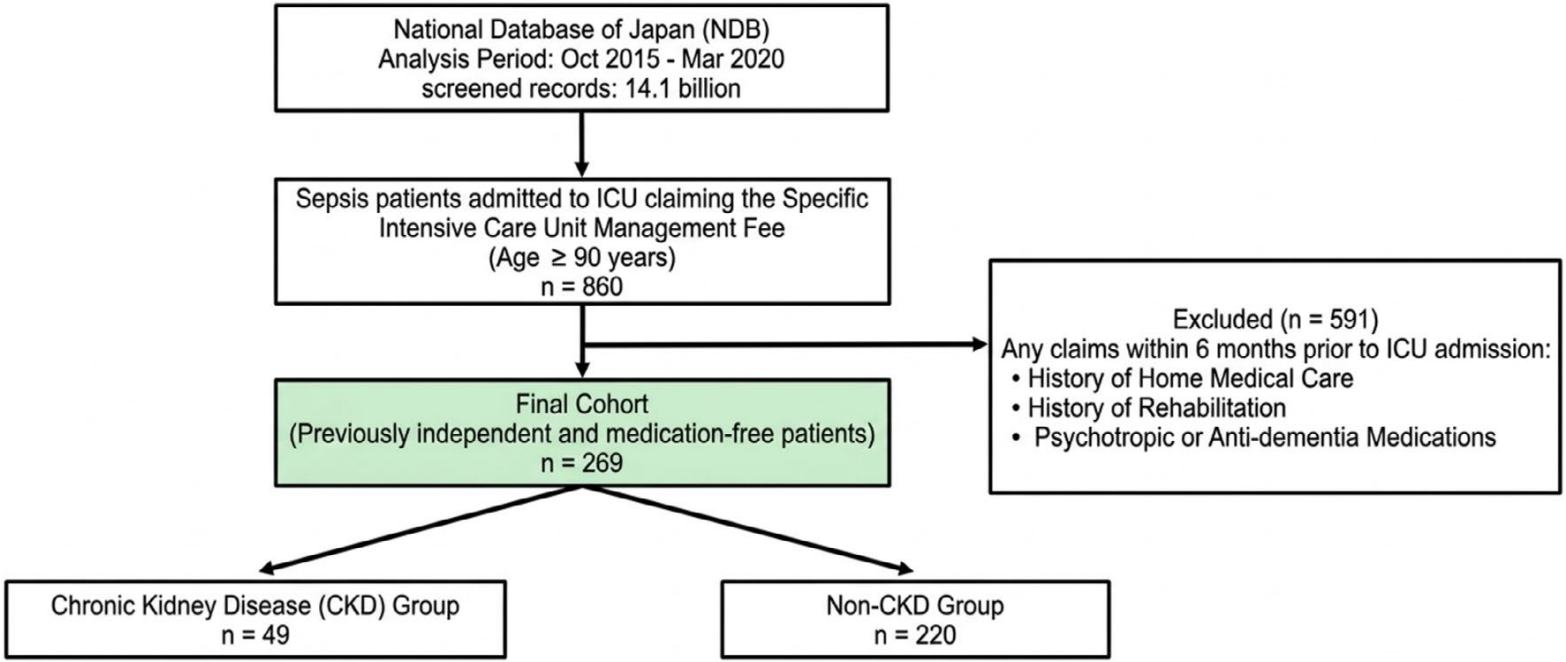
Flowchart of study population selection. From approximately 14.1 billion records, we identified 269 previously independent and medication‐free sepsis survivors. The cohort was stratified into the chronic kidney disease (CKD) group (*n* = 49) and the non‐CKD group (*n* = 220).

From the comprehensive NDB dataset covering the designated period, we initially screened approximately 14.1 billion records to identify 860 potential sepsis candidates aged ≥ 90 admitted to ICUs claiming the specific intensive care unit management fee. After applying our rigorous exclusion criteria to identify previously independent and medication‐free patients (no history of home medical care, rehabilitation or psychiatric/dementia medications), 269 patients were included in the final analysis. This stringent selection process highlights the rarity of truly independent individuals in this age group (Figure 1).

### 3.2 Baseline Characteristics and Comorbidities

Of the 269 previously independent and medication‐free survivors, 49 (18.2%) were diagnosed with pre‐existing CKD. The CKD group had a significantly higher prevalence of vascular comorbidities, including hypertension (masked; *p* = 0.023) and dyslipidaemia (46.9% vs. 30.0%; *p* = 0.029), reflecting a high systemic vascular burden. The implementation rate of CHDF was naturally higher in the CKD group (*p* = 0.012). Importantly, the implementation rate of rehabilitation—a known trigger for PICS—was identical between the groups (51.0% vs. 54.1%; *p* = 0.75), ensuring that the comparison was not confounded by the ‘burden of recovery’.

### 3.3 The ‘Toxic’ Impact: Agitation vs. Dementia

Univariate analysis revealed a striking phenotypic difference in neurological outcomes (Table 1). The CKD group showed a significantly higher rate of antipsychotic initiation (34.7% vs. 19.1%; *p* = 0.022). In stark contrast, the initiation of antidementia drugs was identical between the groups (*p* = 1.000). This discrepancy suggests that the ‘brain failure’ associated with CKD is biologically distinct from Alzheimer's or vascular dementia; it is an acute, hyperactive and likely reversible state. Furthermore, despite the significant mental decline, the transition to home medical care (physical dependence) did not differ significantly (26.5% vs. 32.7%; *p* = 0.50), suggesting that the toxicity of CKD is brain‐specific and does not necessarily correlate with physical frailty.

**TABLE 1:** Baseline characteristics and clinical outcomes of previously independent and medication‐free sepsis survivors aged ≥ 90 years stratified by chronic kidney disease (CKD).

Variable	Total (*N* = 269)	CKD group (*n* = 49)	Non‐CKD group (*n* = 220)	*p*	Odds ratio
Demographics					
Age ≥ 95 years	50 (18.6%)	11 (22.4%)	39 (17.7%)	0.424	1.34
Sex	Masked	Masked	Masked		
Comorbidities					
Hypertension	207 (77.0%)	Masked	Masked	0.023	N/A
Dyslipidaemia	89 (33.1%)	23 (46.9%)	66 (30.0%)	0.029	2.06
Liver dysfunction	50 (18.6%)	14 (28.6%)	36 (16.4%)	0.066	2.04
Diabetes mellitus	147 (54.6%)	30 (61.2%)	117 (53.2%)	0.343	1.39
Ischaemic heart disease	145 (53.9%)	31 (63.3%)	114 (51.8%)	0.157	1.60
Chronic heart failure	159 (59.1%)	34 (69.4%)	125 (56.8%)	0.112	1.72
Malignancy (cancer)	60 (22.3%)	13 (26.5%)	47 (21.4%)	0.450	1.33
DVT	49 (18.2%)	10 (20.4%)	39 (17.7%)	0.684	1.19
Interventions					
Rehabilitation	144 (53.5%)	25 (51.0%)	119 (54.1%)	0.752	0.88
General anaesthesia	89 (33.1%)	14 (28.6%)	75 (34.1%)	0.506	0.77
Mechanical ventilation	91 (33.8%)	15 (30.6%)	76 (34.5%)	0.739	0.84
CVC	91 (33.8%)	20 (40.8%)	71 (32.3%)	0.316	1.45
CHDF	< 10	< 10	< 10	0.012	N/A
Outcomes (mental)					
Any CNS medication	83 (30.9%)	21 (42.9%)	62 (28.2%)	0.059	1.91
Antipsychotics (agitation)	59 (21.9%)	17 (34.7%)	42 (19.1%)	0.022	2.25
Antidepressants	59 (21.9%)	16 (32.7%)	43 (19.5%)	0.056	2.00
Hypnotics (insomnia)	16 (5.9%)	< 10	Masked	0.181	N/A
Neuropathic pain meds	16 (5.9%)	< 10	Masked	0.503	N/A
Dementia meds	11 (4.1%)	< 10	< 10	1.000	N/A
Outcomes (physical)					
Home medical care	85 (31.6%)	13 (26.5%)	72 (32.7%)	0.497	0.74
Concurrent decline in physical and mental autonomy	34 (12.6%)	< 10	Masked	0.793	N/A

*Note:* Data are presented as *n* (%). *p*‐values were calculated using Fisher's exact test.

In accordance with NDB privacy protection regulations, cells containing fewer than 10 patients are suppressed and denoted as ‘< 10’. To prevent secondary disclosure through algebraic reverse‐calculation, corresponding counts in the opposing group are denoted as ‘masked’, and their odds ratios are listed as ‘N/A’. Percentages for these categories are withheld to prevent re‐identification. Fisher's exact test was performed using the unmasked raw data.

Abbreviations: CHDF, continuous haemodiafiltration; CKD, chronic kidney disease; CNS, central nervous system; CVC, central venous catheter; DVT, deep vein thrombosis.

### 3.4 Multivariate Analysis: Confirming the Metabolic Dichotomy

In the primary multivariate model for any CNS medication (Table 2), CKD emerged as a powerful, independent risk factor (OR 2.85; 95% CI 1.30–6.22; *p* = 0.009). This risk was observed after adjusting for the massive impact of rehabilitation (OR 11.63; *p* < 0.001) and age. To confirm the ‘agitation phenotype’, we performed a secondary multivariate analysis specifically for antipsychotics (Table 3). In this model, the impact of CKD became even more pronounced (OR 3.22; 95% CI 1.46–7.11; *p* = 0.004), confirming that CKD is a specific and potent driver of ‘toxic agitation’. Crucially, liver dysfunction maintained its trend as a ‘protective’ or neutral factor (OR 0.57–0.71), statistically illustrating the ‘metabolic dichotomy’—the kidney attacks (OR > 1), while the liver shields (OR < 1).

**TABLE 2:** Multivariate logistic regression analysis identifying independent predictors of new CNS medication initiation in sepsis survivors aged ≥ 90 years.

Variable	OR	95% CI	*p*
The toxic filter (risk)			
CKD	2.85	1.30–6.22	0.009
The trigger (risk)			
Rehabilitation	11.63	5.43–24.91	< 0.001
The shield (protective)			
Liver dysfunction	0.71	0.30–1.68	0.437
Adjusted covariates			
General anaesthesia	1.20	0.64–2.26	0.568
Age ≥ 95 years	0.99	0.44–2.21	0.971

*Note:* The primary outcome is defined as the new initiation of any CNS‐active medications (antipsychotics, antidepressants, anxiolytics, antidementia drugs or hypnotics) within 6 months after ICU admission.

Model Specification: The multivariate logistic regression model was adjusted for all variables listed in the table (CKD, rehabilitation, liver dysfunction, general anaesthesia and age ≥ 95 years). *p* < 0.05 is considered statistically significant.

Abbreviations: CI, confidence interval; CKD, chronic kidney disease; CNS, central nervous system; OR, odds ratio.

**TABLE 3:** Secondary multivariate analysis identifying independent predictors of antipsychotic initiation (‘Toxic Agitation’) in sepsis survivors aged ≥ 90 years.

Variable	OR	95% CI	*p*
The toxic filter			
CKD	3.22	1.46–7.11	0.004
Other factors			
Rehabilitation	9.23	3.86–22.07	< 0.001
Liver dysfunction	0.57	0.21–1.51	0.256
General anaesthesia	1.39	0.71–2.73	0.332
Age ≥ 95 years	1.10	0.47–2.61	0.823

*Note:* The secondary outcome is defined strictly as the new initiation of antipsychotic medications within 6 months after ICU admission, serving as a clinical surrogate for severe agitation or delirium (toxic agitation).

Model Specification: The multivariate logistic regression model was adjusted for all variables listed in the table (CKD, rehabilitation, liver dysfunction, general anaesthesia and age ≥ 95 years) to ensure independence from the ‘rehabilitation trigger’ and the ‘liver shield’. *p* < 0.05 is considered statistically significant.

Abbreviations: CI, confidence interval; CKD, chronic kidney disease, OR, odds ratio.

### 3.5 Robustness of Findings

To rule out the possibility that the agitation was caused by the acute stress of dialysis (e.g., dialysis disequilibrium syndrome), we performed sensitivity analyses excluding patients who underwent renal replacement therapy (CHDF). The results remained consistent (OR 3.21; 95% CI 1.41–7.33; *p* = 0.006), confirming that the risk is driven by chronic renal dysfunction itself and the accumulation of endogenous toxins, not by the acute dialysis procedure.

## 4 Discussion

### 4.1 The ‘Toxic Filter’ Theory: Uremic Encephalopathy Revisited

Our findings establish CKD as a distinct and powerful driver of neuro‐psychiatric decline in nonagenarians. In our univariate analysis, the association between CKD and overall CNS medication initiation was marginal (*p* = 0.059). However, multivariate adjustment robustly unmasked this risk (OR 2.85; *p* = 0.009). This phenomenon of negative confounding highlights that the true neurotoxic impact of CKD is only fully realised when adjusted for the protective ‘brake’ of liver dysfunction and the dominant ‘trigger’ of intensive rehabilitation. The most notable finding is the specific nature of the decline: CKD increased the risk of Antipsychotics (OR 3.22) far more than it did for general CNS medications, with zero increase in dementia medications. This strongly supports the hypothesis of ‘toxic agitation’. In these patients, the ‘filter’ is broken. Uremic toxins (e.g., indoxyl sulphate, guanidino compounds, homocysteine) and active metabolites of medications accumulate [13]. For example, morphine‐6‐glucuronide (an active metabolite of morphine) and alpha‐hydroxymidazolam (an active metabolite of midazolam) rely heavily on renal excretion [14, 15]. In nonagenarians, who likely possess a leaky blood–brain barrier due to sepsis and age, these toxins readily enter the CNS. Once in the brain, they cause direct neurotoxicity, neurotransmitter imbalance (increasing glutamatergic excitatory transmission) and paradoxical excitation [16]. This state, classically known as ‘uremic encephalopathy’, masquerades as psychiatric illness or ‘difficult behaviour’ but is fundamentally a metabolic intoxication [10].

### 4.2 Metabolic Dichotomy: Shield vs. Spear

This study completes the ‘metabolic’ narrative of our research series, offering a unified theory of organ dysfunction in patients aged ≥ 90 years.

1. *The liver (the shield)*: A failing liver slows drug clearance and systemic metabolic rate. In the context of PICS and iatrogenic interventions, this paradoxically prevents ‘overdose’ and protects the brain from the metabolic stress of active recovery. It acts as a physiological ‘brake’ or ‘Shield’ [1].
2. *The kidney (the spear)*: A failing kidney stops excretion. This turns the body into a closed loop where waste products and toxins continuously cycle and accumulate. It acts as a **‘**spear**’** that actively assaults the brain.

### 4.3 Clinical Implications: ‘Renal Protection is Brain Protection’

The recognition of this dichotomy necessitates a paradigm shift in clinical management for nonagenarians.

*1. Re‐evaluating rehabilitation*: For CKD patients, the standard ‘early mobilisation and rehabilitation’ strategy poses a double threat. As shown in our third report, rehabilitation generates metabolic waste and stress [7]. If the ‘filter’ (kidney) is clogged, this stress has nowhere to go, leading to explosive agitation. Rehabilitation in CKD patients must be ‘renal‐sparing’—gentle, paced, and meticulously monitored for metabolic load and hydration status.

*2. Management of agitation*: The reflex response to agitation in CKD patients is often *more* sedation (e.g., benzodiazepines or antipsychotics). However, our data suggests this is dangerous. Since the agitation is driven by accumulation, adding more drugs that require renal clearance only fuels the fire. The correct strategy should be ‘active washout’—enhancing clearance through adequate hydration or early, aggressive renal replacement therapy to remove the ‘toxic’ burden before resorting to chemical restraints [17].

### 4.4 Limitations

This study has limitations inherent to administrative claims data. Crucially, we lacked detailed laboratory parameters (such as serum creatinine, estimated GFR, AST, ALT or bilirubin levels). Consequently, we could not provide specific clinical cut‐off values or classify the exact pathophysiological nature (e.g., enzyme abnormality vs. synthetic dysfunction) of the renal and liver dysfunctions. These conditions were defined solely based on registered ICD‐10 codes. In claims data like the NDB, early‐stage, asymptomatic organ dysfunction may be undercoded. Therefore, our ‘CKD group’ and ‘liver dysfunction group’ likely represent patients with clinically significant, moderate‐to‐severe impairment that required physician attention. Far from invalidating our findings, this actually strengthens our ‘toxic filter’ hypothesis: only clinically significant filtration failure causes sufficient neurotoxin accumulation to drive toxic agitation. Furthermore, the consistency of the results across multivariate and sensitivity analyses, and the biological plausibility of the ‘Toxic Filter’ mechanism, strengthen our conclusions. Future prospective studies should correlate serum toxin levels with agitation scores in this population.

In sepsis survivors aged ≥ 90 years, the kidney is the guardian of sanity. Its failure turns the body into a toxic reservoir, driving severe agitation that mimics psychiatric illness. To protect the brain of nonagenarians, clinicians must look beyond the brain itself and protect the filter. “Renal Protection is Brain Protection.”

## Funding

This work was supported by Japan Society for the Promotion of Science (JSPS) KAKENHI (Grant Number JP21K09088).

## Ethics Statement

The study protocol was approved by the Ethics Committee of Osaka Medical College (currently Osaka Medical and Pharmaceutical University), Osaka, Japan (Approval No. 2871‐2). The study was carried out in accordance with The Code of Ethics of the World Medical Association (Declaration of Helsinki). All methods were carried out in accordance with relevant guidelines and regulations.

## Consent

Given the retrospective nature of the study and the use of anonymized administrative data, the requirement for individual informed consent was waived in accordance with the Ethical Guidelines for Medical and Health Research Involving Human Subjects in Japan.

## Conflicts of Interest

The authors declare no conflicts of interest.

## Data Availability

The data that support the findings of this study are available from the Ministry of Health, Labour and Welfare (MHLW) of Japan, but restrictions apply to the availability of these data, which were used under license for the current study, and so are not publicly available. Data are strictly controlled under the ‘Act on Assurance of Medical Care for Elderly People’ to protect personal information and cannot be shared by the authors. Researchers who meet the criteria for data access may apply directly to the MHLW for access to the NDB data.
